# Correlation of CSF- and MRI-Biomarkers and Progression of Cognitive Decline in an Open Label MCI Trial

**DOI:** 10.14283/jpad.2018.5

**Published:** 2018-02-13

**Authors:** L.K. Joachim, L. Frölich, E. Rüther, J. Wiltfang, W. Maier, J. Kornhuber, C. Bauer, I. Heuser, Oliver Peters

**Affiliations:** 1 Department of Psychiatry, Charité – Universitätsmedizin Berlin, corporate member of Freie Universität Berlin, Humboldt-Universität zu Berlin, and Berlin Institute of Health (BIH), Campus Benjamin Franklin, Berlin, Germany; 2 Department of Geriatric Psychiatry, Central Institute of Mental Health, Medical Faculty Mannheim, University of Heidelberg, Mannheim, Germany; 3 Department of Psychiatry and Psychotherapy, University Medical Center Göttingen, Göttingen, Germany; 4 Department of Psychiatry, University Bonn, Bonn, Germany; 5 Department of Psychiatry, Friedrich-Alexander-University of Erlangen-Nuremberg, Erlangen, Germany; 6 MicroDiscovery GmbH, Berlin, Germany; 7 German Center for Neurodegenerative Diseases (DZNE) Göttingen, Göttingen, Germany; 8 German Center for Neurodegenerative Diseases (DZNE) Bonn, Bonn, Germany; 9 German Center for Neurodegenerative Diseases (DZNE) Berlin, Berlin, Germany; 10 Department of Psychiatry and Psychotherapy, Charité – Universitätsmedizin Berlin, Campus Benjamin Franklin, Hindenburgdamm 30, 12203, Berlin, Germany

**Keywords:** Cerebrospinal fluid, Alzheimer's disease, amyloid β1-42, total-Tau, hippocampal atrophy, galantamine, AChE-I

## Abstract

BACKGROUND: In several randomized controlled trials (RCT) acetylcholinesterase-inhibitors (AChE-I) were tested in patients with mild cognitive impairment (MCI) but were ineffective in delaying disease progression as determined by neuropsychological testing only. Here we present data from an open label observational extension of a multicenter RCT in order to assess if biomarkers are providing useful additional information about a drug's efficacy. We followed 83 amnestic MCI patients and performed correlational analyses of Aβ 1–42 and total-Tau in the cerebrospinal fluid (CSF), hippocampal and amygdala volume at baseline, the total duration of blinded and open label AChE-I treatment and the outcome 24 months after inclusion into the RCT. Twelve out of 83 amnestic MCI (14%) had progressed to Alzheimer's disease (AD). Overall, worsening and disease progression as measured by the Alzheimer's Disease Assessment Scale - cognitive subscale (ADAS-cog), Alzheimer's Disease Cooperative Study - Activities of Daily Living (ADCS-ADL) and Clinical Dementia Rating (CDR) did not correlate with the duration of AChE-I treatment. However, a specific multidimensional biomarker profile at baseline indicated more reliably than cognitive testing alone progression to AD. We conclude that pharmacological RCTs testing symptomatic treatment effects in MCI should include biomarker assessment.

## Introduction

Mild cognitive impairment (MCI) represents a heterogeneous condition comprising several clinical subtypes. Some MCI, especially those in prodromal stages of Alzheimer's disease (AD), carry a substantial risk of developing dementia (1, 2). Thus, treatment strategies to delay or even prevent progression from MCI to dementia are a major goal. There is currently no established regimen to successfully treat symptoms of MCI. Clinical trials in MCI have tested acetylcholinesterase-inhibitors (AChE-I) with the aim to improve cognitive function and to delay disease progression (3, 4, 5, 6, 7, 8, 9). However, lasting symptomatic improvement or a delay in disease progression could not be shown (donepezil (3, 4, 5), rivastigmine (6), galantamine (7, 8)). Similarly, a randomized controlled trial (RCT) of memantine in MCI patients was unable to significantly improve cognition, but was suggestive for a positive effect on attention and information processing speed [9, 10]. Detailed post hoc analyses to identify the reasons why antidementia drugs have failed to be efficacious in MCI have rarely been performed (11). Thus, we performed a retrospective correlational analysis in a recent RCT to address this issue. We used the data from a multicenter study within the German Dementia Competence Network (DCN) that had tested the safety and efficacy of a combination of galantamine plus memantine in patients with MCI (MCI-COMBI). At study inclusion cerebrospinal fluid (CSF) - and neuroimaging-markers had been obtained (12, 13).

Widely accepted biomarkers of AD pathology are CSF β-amyloid 1-42 (ACSF β 1–42), CSF total-Tau and structural MRI measures of atrophy. Low concentrations of CSF Aβ 1–42 correlate with brain Aβ-plaque load and Aβ neuropathology at autopsy (14). The ratio of reduced CSF Aβ1–42 and increased CSF-Tau is considered to be a signature representing AD neuropathology (15). Although increased CSF-Tau is not specific for AD, elevated Tau correlates with disease progression (16). The magnitude of cerebral atrophy can be assessed by reduced hippocampal and amygdala volume using MRI volumetry. Hippocampal atrophy indicates progression of neurodegeneration and is tightly correlated with both neuropathological measures of tangle load and cognitive symptoms (17). Thus, CSF- and MRI-measurements reflect loss of neuronal integrity and functional and cognitive decline (18).

In this paper we report on a post-hoc analysis of the association between biomarker parameters, symptomatic effects and disease progression seen in our original MCI-COMBI study, described in detail elsewhere (13).

## Methods

### Participants and study procedures

Patients with MCI were included into the MCI-COMBI trial, which was initially planned as a randomized two years, double-blind, placebo-controlled multicenter study. The study design, recruitment, inclusion and exclusion criteria as well as methods of biomarker measurements (i.e. CSF analytics and MRI techniques) were previously described in detail (12, 13). The double-blind treatment in the MCI-COMBI study had been prematurely stopped upon advice of the steering committee, due to an imbalance of serious adverse events in the treatment and the placebo group in two other large multinational MCI studies testing the effect of galantamine in MCI (Gal Int-11/-18). At this point, in our study, 232 patients with amnestic MCI (placebo n=79, galantamine n=75 or galantamine plus memantine n=78) had already been randomized and had received study medication for at least two weeks and for about one year at the most. An adapted study procedure had then been implemented and study medication had been tapered over four weeks. The results of the blinded treatment and controlled discontinuation of the study have already been reported (13).

After discontinuation procedure the open label extension (OLE) of the MCI-COMBI trial was started, and it was decided on an individual level whether or not treatment with an AChE-I was initiated. All participants were invited to perform a reassessment comprising the Alzheimer's Disease Assessment Scale - cognitive subscale (ADAS-cog), the Alzheimer's Disease Cooperative Study - Activities of Daily Living (ADCS-ADL) and the Clinical Dementia Rating — sum of boxes (CDR-SB) to investigate cognitive worsening and disease progression. In addition, the overall cumulative treatment time was calculated by adding the number of days patients had received any AChE-I either within the aborted RCT (galantamine only) or within the OLE (galantamine, rivastigmin or donepezil). The maximum cumulative treatment time therefore was 728 days and comprised the period the patients received study medication plus antidementive treatment during the open label extension. MCI patients, who received placebo during the trial and who were left without antidementive treatment afterwards, ended up with a total treatment time of zero. Since no differences were detected between AChE-I monotherapy and memantine-add-on in the blinded part of the MCI-COMBI trial, memantine as part of the study medication was neglected in further analyses. None of the patients who were followed 24 months in the OLE received memantine, since memantine is approved in Germany for moderate to severe AD only.

Out of 83 patients who were followed in the OLE, 39 underwent a lumbar puncture at baseline, 42 had a MRI volumetry and 21 had both. Post-hoc correlational analyses of these parameters were done to explore the reliability of these biomarkers to reflect disease progression.

### Ethical Considerations and safety analyses

The original and the adapted study design and the protocols of the MCI-COMBI study had been approved by the Ethics Review Board of Charité Berlin and were reaffirmed by the Ethics Committees at each individual center. All subjects had given written informed consent to the initial and to the modified study protocol. All procedures were in accordance with ethical standards of the responsible committees on human experimentation, with the revised Helsinki Declaration and with Good Clinical Practice guidelines.

### Statistical Analyses

To compare outcome variables between baseline and follow-up we used the Wilcoxon rank sum test. To analyze whether the subsample (n=83) showed the same distributions as the whole sample (n=232), the Wilcoxon rank sum test was applied. For correlational analyses we used Pearson's correlation coefficient (annotated as cor). Linear regression analyses were performed in order to test the significance of the correlation. The p-values of the correlation-scatterplots were calculated with ANOVA. Data were analyzed using IBM SPSS for Mac (version 22) and R version 3.2.4. Statistical tests were two-tailed, and an α-level below 0.05(*) was considered significant.

## Results

**Figure 1:**
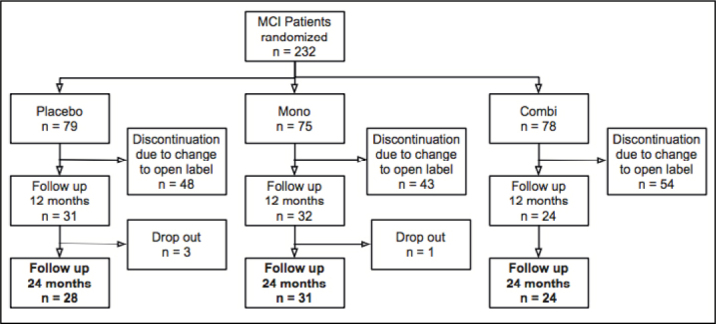
Study flow chart. Mono: Galantamine only; Combi: Galantamine and Memantine

Only eighty-three of the 232 patients from the original MCI-COMBI study participated in the OLE and performed a complete clinical and neuropsychological reassessment after 24 months. The most common reason for patients not to participate in the OLE was the disappointment about the premature stop of the RCT and the unforeseen changes in the study procedure. Only very few patients were unable to visit the study center for other reasons and were lost to follow up (Figure 1). Comparing the smaller follow up-group with the larger original MCI-COMBI group revealed no differences with respect to age, gender, education, neuropsychological test performance and biomarker profiles. A comparison of neuropsychological test performances (ADAS-cog) and biomarker profiles (Aβ 1-42, total-Tau, phospho- Tau, hippocampal and amygdala volume) is shown in supplementary Figure 1.

### Outcome at the end of the open label extension 24 months after baseline

Three outcome variables reflecting cognitive performance (ADAS-cog), impairment of daily living (ADCS-ADL) and disease progression (CDR-SB) were analyzed. The mean of ADAS-cog (11.3 ± 6.7 at baseline) remained unchanged (11.2 ± 6.7 at 24 months, p = 0.36). The mean of ADCS-ADL-sum worsened slightly but insignificantly from baseline (50.3 ± 5.9) to 47.1 ± 10 after 24 months (p = 0.11); the same was true for CDR-SB (1.34 ± 0.9 at baseline; 1.77 ± 1.8 after 24 months; p = 0.87). Two years after baseline 12 out of 83 MCI patients (14%) had progressed to dementia and fulfilled the diagnostic criteria for dementia due to Alzheimer's disease.

### Treatment did not prevent cognitive and functional decline as measured by the ADAS-cog, ADCS-ADL and CDR-SB

MCI-to-AD converters had received treatment for at least 238 days. Eight out of 12 converters had been treated for the maximum period of 24 months (728 days). The total cumulative treatment time of converters was longer than for non-converters (630 ± 174 vs. 211 ± 233 days). Obviously ADAS-cog scores increased in MCI-to-AD converters: the ADAS-cog score changed from 14.2 ± 5.7 at baseline to 19.4 ± 8.6 after 24 months.

**Table 1:** Descriptive data of baseline characteristics are shown as mean with standard deviation

Baseline characteristics	RCT (n=232-83)	OLE (n=83)
ADAS-cog	11.24 ± 4.95	11.47 ± 4.87
Amyloid β 1–42 (pg/ml)	899.61 ± 336.81^a^	1071.56 ± 487.01^e^
Total-Tau (pg/ml)	499.30 ± 370.58^b^	411.41 ± 239.00^f^
Phospho-Tau (pg/ml)	69.35 ± 37.35^c^	65.28 ± 31.12^g^
Hippocampal volume left (mm^3^)	1845.23 ± 415.19^d^	1977.94 ± 509.35^h^
Hippocampal volume right (mm^3^)	1764.68 ± 572.75^d^	1988.53 ± 530.05^h^
Amygdalar volume left (mm^3^)	620.14 ± 361.00^d^	526.03 ± 141.92^h^
Amygdalar volume right (mm^3^)	531.90 ± 131.09d	558.049 ± 142.53h


RCT: Randomized controlled trial; OLE: Open label extension; MMSE: Mini-Mental State Examination; ADAS-cog: Alzheimer's Disease Assessment Scale - cognitive subscale; ADCS-ADL: Alzheimer's Disease Cooperative Study-Activities of Daily Living; CDR: Clinical Dementia Rating. Superscript CSF- and MRI biomarkers: a (n= 56), b (n = 55), c (n = 57), d (n=48); e (n = 39), f (n = 39), g (n= 39) and h (n= 42).

**Figure 2:**
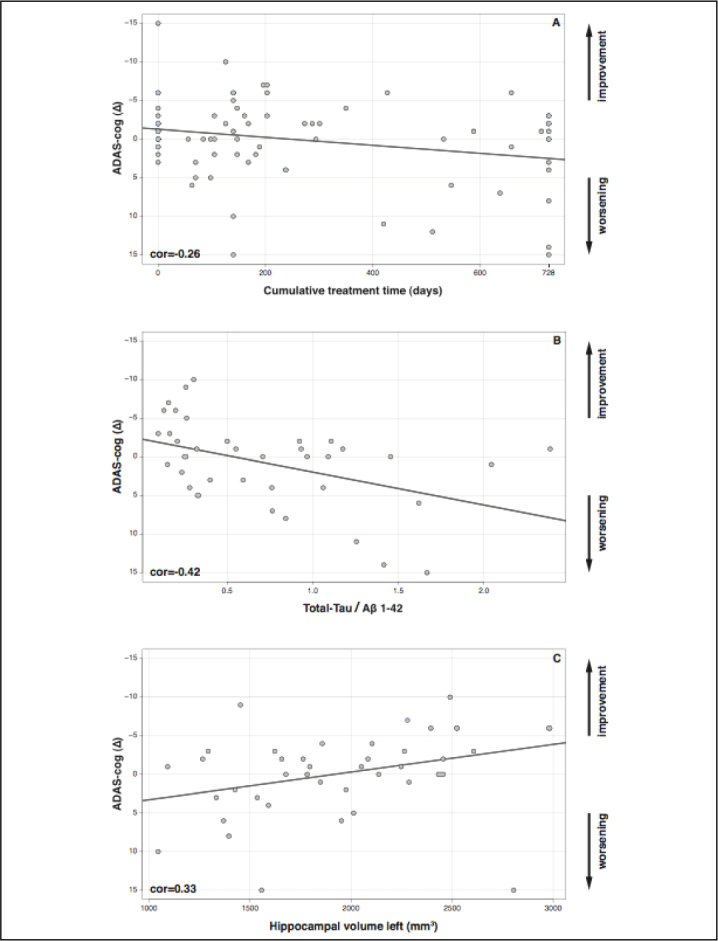
Changes in ADAS-cog (Δ) from baseline to 24-months follow-up. (A) ADAS-cog and Cumulative treatment (p = 0.024), (B) total-Tau/β 1-42 ratio (p = 0.0043) and (C) left hippocampal volume (p = 0.03).

In the sample of these 83 patients a negative correlation between the cumulative treatment period and cognitive changes was observed (p = 0.024, cor = - 0.26, Figure 2A). Also, treatment did not prevent worsening of ADCS-ADL and CDR-SB scores (Δ ADCS-ADL, p = 0.006, cor = − 0.33; Δ CDR-SB, p = < 0.001, cor = − 0.41; for more information see also supplementary Figure 3). For further analyses we focused on the relationship between biomarkers and cognition as measured by the ADAS-cog, because previous correlational analyses had revealed AChE-I to have at least a temporary effect on ADAS-cog scores but not on ADCS-ADL or CDR-SB scores (13).

### CSF-biomarkers at baseline are tightly associated to changes in cognitive function over time

A significant decrease in cognitive performance was detected in those patients with MCI who had low levels of Aβ 1–42 in comparison to patients with higher levels of Aβ 1–42 (suppl. Figure 2A; p = 0.005, cor = 0.44). High levels of total-Tau were associated with a significant cognitive deterioration (suppl. Figure 2B; p = 0.006, cor = − 0.43). The total-Tau/Aβ 1–42 ratio reflects the overall relationship of CSF-biomarkers with cognitive worsening (Figure 2B): high ratios corresponded to an increase in ADAS-cog scores (Figure 2B; p = 0.004, cor = − 0.42).

### Hippocampal and amygdala volume at baseline are associated with changes in cognitive function over time

Patients with MCI whose cognitive performance deteriorated had lower left hippocampal volumes (Figure 2C; p = 0.03, cor = 0.33), whereas patients with higher volumes showed even an improvement in neuropsychological testing over time. The right hippocampal (p = 0.11, cor = 0.25) and the amygdala volumes displayed the same tendency but were not statistically significantly correlated to cognitive changes (left amygdala: p = 0.15, cor = 0.22; right amygdala: p = 0.39, cor = 0.14).

Additionally, we analyzed the relationship between APOE-status and cognitive worsening. The loss of cognitive function (ADAS-cog) was not significantly correlated with the presence or absence of Apo E ε4 homo- or heterozygoty (p = 0.054, cor: −0.22).

## Discussion

The purpose of our post-hoc, retrospective analyses was to better understand the relationship between biomarker measurements and progression of cognitive performance in MCI patients treated with an AChE-I. Our major finding was that those patients with a more AD-like CSF profile (low Aβ 1–42 level, high total-Tau level) and smaller left hippocampi at baseline, declined faster and more profoundly so that a possible benefit of AChE-I was undetectable. Surprisingly, the total cumulative treatment time was even longer in MCI to AD-converters than in non-converters, most likely due to early prescription of antidementia drugs in the OLE to MCI progressors. Although MCI with AD-typical biomarkers might have had some benefit from AChE-I treatment, comparison of correlation coefficients in our study suggested, that this effect was blunted by the dynamics of neurodegeneration as reflected by CSF- and MRI-biomarkers.

### Biomarkers at baseline predict reliably clinical outcome correlate with cognitive worsening

Our MCI-COMBI trial, as all other MCI studies that had tested AChE-I in RCTs (3, 4, 5, 6, 7, 8), had selected patients at risk based on a thorough neuropsychological testing to characterize the typical amnestic cognitive deficits associated with the prodromal stage of Alzheimer's dementia (12, 13). In our study CSF-biomarker and MRI-volumetry measurements were additionally performed as part of an addendum at baseline, but were not part of the diagnostic workup. Our post-hoc analyses reveal that CSF- and MRI-biomarkers at baseline significantly correlated with cognitive worsening over time. Regarding the prognostic value of biomarkers for AD pathology and their obvious reflection of disease progression our results are in line with previous studies. It has been shown numerously that the relative risk of progression to dementia is considerably higher in patients with MCI who have typical AD biomarker measurements, namely increased total-Tau and lowered Aβ 1–42 (15, 16, 17, 18, 19). While Aβ species have been identified as Alzheimer's disease markers, CSF tau levels are mainly correlated with progression of neurodegeneration. For example, CSF total-Tau and phospho-Tau have been found to be significantly higher in early converters versus late converters (16). In a long-term follow-up study the baseline Aβ 1–42/p-Tau ratio predicted the conversion to AD within 9.2 years with a sensitivity of 88% and a specificity of 90%. The positive predictive value in this study was 91% and the negative one was 86% (20).

MRI analyses by Teipel et al. demonstrated an association between ADAS-cog score and both the left hippocampus and bilateral amygdala volumes. Global cognitive scales like MMSE, ADAS-cog and CDR-SB correlated with volumetric measures (21). Similar to our results, a placebo-controlled study recently observed that the baseline hippocampal volume was a significant predictor for cognitive decline in amnestic MCI patients. The impact was independent of AChE-I treatment for a period of 12 months and a 6 months open label follow up (22).

### AChE-I do not prevent patients with MCI to progress to AD

Treatment with any AChE-I in patients with MCI has failed to delay progression to dementia (3, 4, 5, 6, 7, 8), but detailed analyses aiming to clarify the reasons are rare. One might speculate MCI may have only a slight cholinergic deficit that cannot be relieved by AChE-I. In earlier analyses of the MCI-COMBI trial we were able to show that only MCI patients with prodromal AD, validated by a typical CSF biomarker profile, but not MCI due to other etiologies had a slight and transitory cognitive benefit after short-term antidementia treatment (13). Very recently, re-analyzing data from one of the large MCI trials, “false positive” MCI subjects with normal performance in more extensive cognitive testing were detected. These “false positive” MCI have low rates of progression to AD and may have the potential to weaken or obscure meaningful findings. Removing these false positive MCI subjects from the cohort significantly strengthened the apparent beneficial effects of donepezil (4, 11).

In summary, the value of biomarkers validating AD pathology as a prerequisite for clinical trials in MCI has increasingly been recognized within the last years. Our results add to the demand of controlling for biomarker heterogeneity in clinical trials and that they should be part of the inclusion criteria in any clinical trials testing symptomatic or disease-modifying effects of antidementia drugs. Our findings underline along with others that CSF- and MRI-biomarkers help to predict disease progression, conversion from MCI to AD and help to define neuropathological disease severity (15, 23).

### Limitations of the study

The data shown represent post-hoc exploratory correlation analyzes in a rather small sample of MCI patients. The premature stop of the double-blind placebo controlled trial caused a substantial drop-out rate. Therefor all conclusions on the results are obviously limited. However, we do feel that the post-hoc analyses, as limited as they may be, underscore the necessity to include biomarker sampling in RCTs of antidementia drugs.

*Acknowledgments:* This study has been supported by a grant from the German Federal Ministry of Education and Research (BMBF): Kompetenznetz Demenzen (01GI0420). Additional funding related to the randomized clinical trials came from Janssen-Cilag and Merz Pharmaceuticals. The latter funds were exclusively used for personnel, pharmaceuticals, blistering and shipment of medication, monitoring and as capitation fees for recruiting centers. We wish to thank all patients and their caregivers, as well as all coworkers in the cooperating centers who participated in the trial.

*Author Contributions:* Study concept and design: Heuser, Frölich, Peters. Acquisition, analysis, or/and interpretation of data: All authors. Drafting the manuscript: Joachim, Heuser, Peters. Critical revision of the manuscript for important intellectual content: All authors. Final approval of the version to be published: All Authors. Agreement to be accountable for all aspects of the work in ensuring that questions related to the accuracy or integrity of any part of the work are appropriately investigated and resolved: All Authors.

## Electronic supplementary material

Supplementary material, approximately 109 KB.
