# Supplementary material for: Correlation of CSF- and MRI-Biomarkers and Progression of Cognitive Decline in an Open Label MCI Trial
Source: J Prev Alzheimers Dis. 2018 Feb 13;5(3):202–6. doi: 10.14283/jpad.2018.5 (PMC12280704; doi:10.14283/jpad.2018.5)
Supplement: Supplementary file 1 — Supplementary material, approximately 109 KB. [file mmc1.pdf]

Suppl. Figure 1: Comparison of baseline- and follow up- cohort. Group I (n = 232-83): Randomized controlled trial; Group II (n = 83): Open label extension investigated at 24 month follow up. No significant differences between Group I and II.

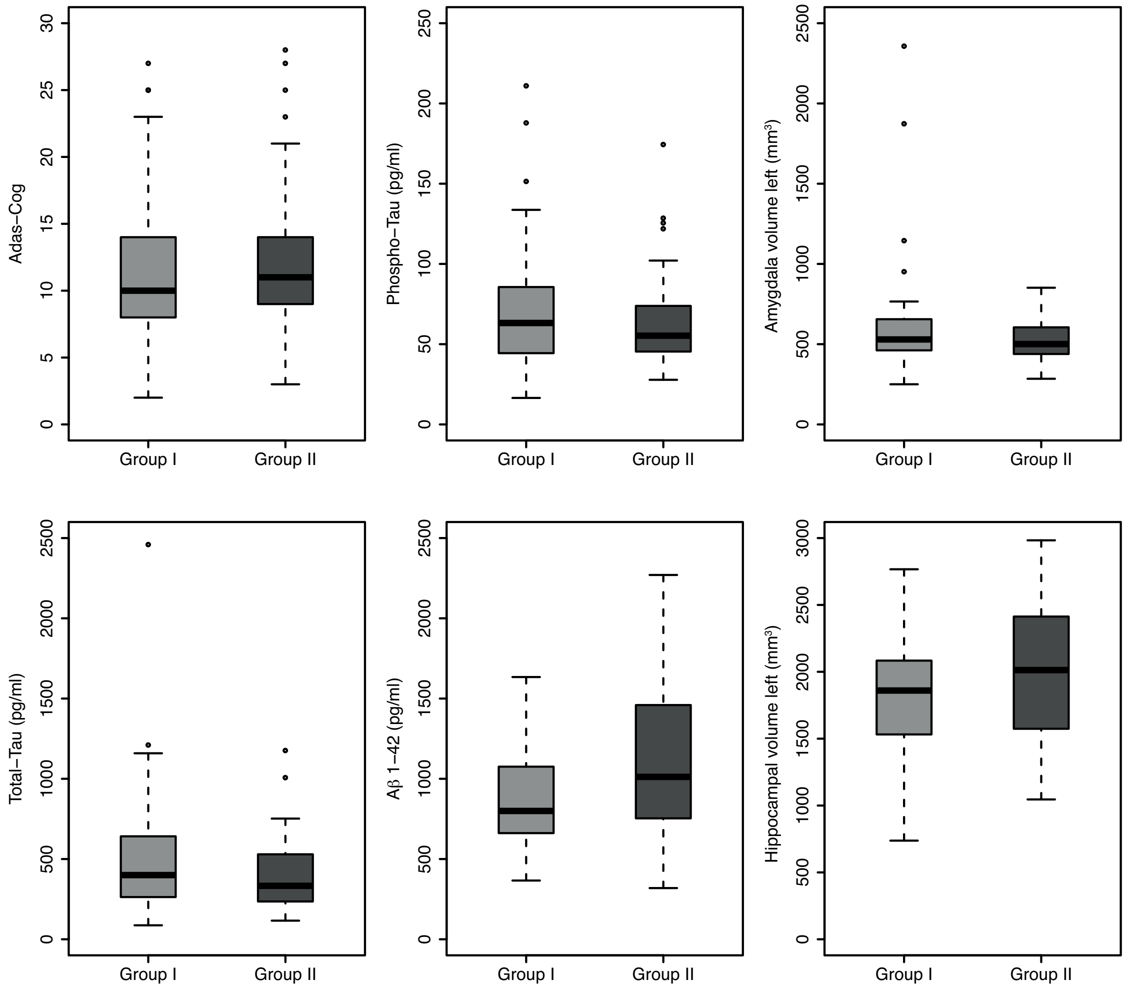

Supplementary Figure 1

Suppl. Figure 2: Changes in ADAS-cog (Δ) from baseline to 24-months follow-up. (A) ADAS-cog and Aβ 1-42 (p = 0.0045) and (B) ADAS-cog and total-Tau (p = 0.0063).

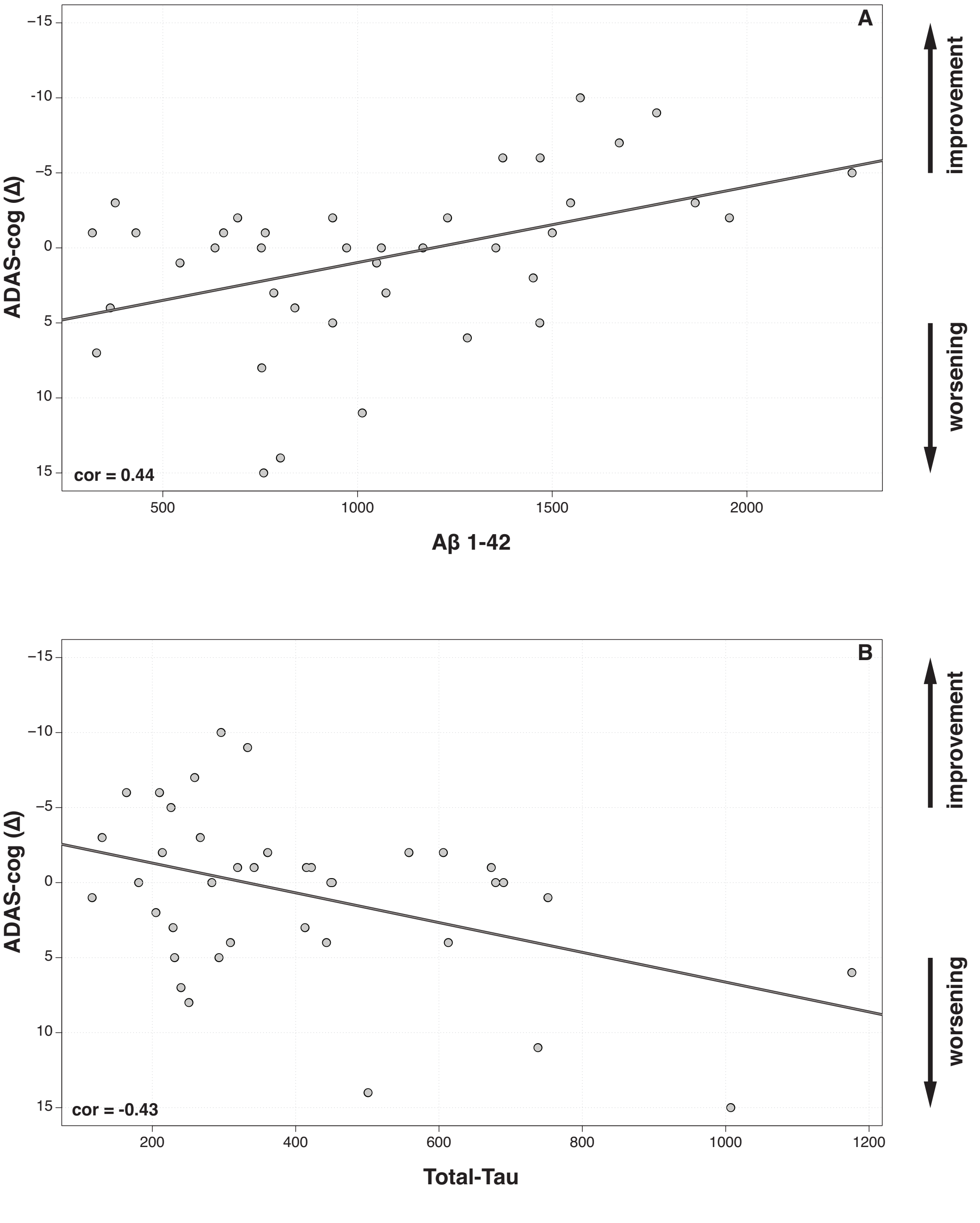

Supplementary Figure 2

<u>Suppl. Figure 3</u>: Changes from baseline to 24-months follow-up. (A) in ADCS-ADL (Δ) and Cumulative treatment ($p = 0.006$), (B) in CDR-SB (Δ) and Cumulative treatment ($p = < 0.001$).

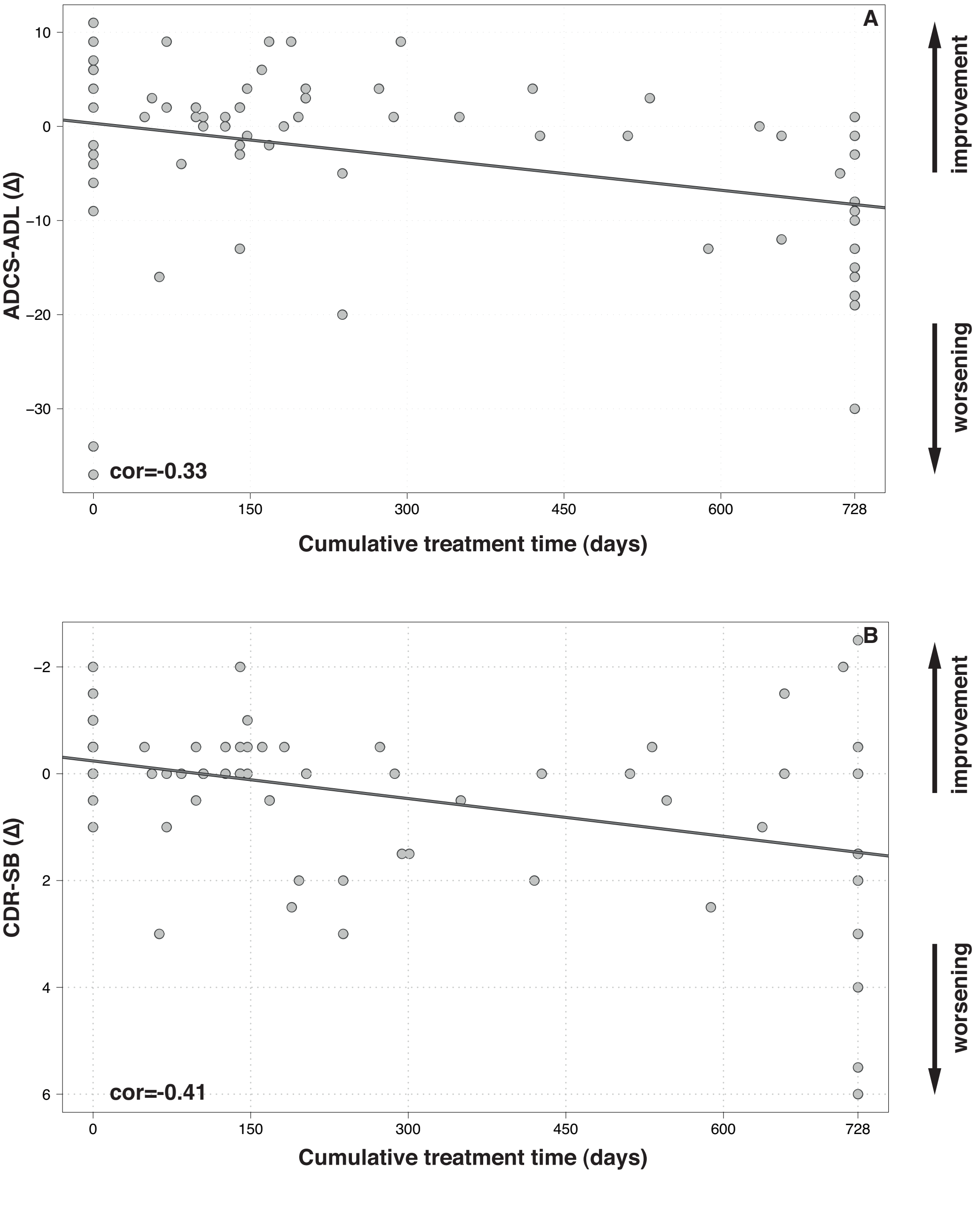

Supplementary Figure 3
